# Assessing Community-Based Injury Prevention Services in U.S. Children's Hospitals

**DOI:** 10.3934/publichealth.2014.4.199

**Published:** 2014-10-31

**Authors:** Nancy L. Weaver, Victoria Kortlandt, Janice Williams, Keri Jupka, Trent D. Buskirk, Salwa Maalouf, Stacy Biddinger, Nancy Hanson, Karen Seaver Hill

**Affiliations:** 1 Department of Behavioral Science and Health Education, College for Public Health & Social Justice, 3545 Lafayette Avenue, Saint Louis University, St. Louis, MO 63104, USA; 2 Department of Biostatistics, College for Public Health & Social Justice, 3545 Lafayette Avenue, Saint Louis University, St. Louis, MO 63104, USA; 3 Department of Emergency Medicine, Carolinas Medical Center, Charlotte NC 28232, USA; 4 College for Public Health & Social Justice, 3545 Lafayette Avenue, Saint Louis University, St. Louis, MO 63104, USA; 5 Children's Hospital Association, Washington, DC 20005, USA

**Keywords:** injury prevention, community outreach, community health needs assessment, pediatrics, children's hospitals

## Abstract

**Objective:**

Not-for-profit hospitals are required to meet federal reporting requirements detailing their community benefit activities, which support their tax-exempt status. Children's hospitals have long provided community injury prevention (IP) programming and thus can inform public health outreach work in other areas. This work describes IP programming as a community service offered by children's hospitals in the U.S.

**Methods:**

The IP specialist at 232 US-based member institutions of the Children's Hospital Association were invited to complete an assessment of their hospital's IP outreach programming.

**Results:**

47.7 percent of hospitals request financial data from IP programming for tax reporting purposes. Almost all offer injury prevention (IP) services; the majority are in the community (60.3%) and 34.5% are hospital-based. Most IP units are independent (60.3%) and 71.8% are responsible for their own budgets.

**Conclusions:**

By integrating dissemination and implementation sciences and community health needs assessments, these findings can help advance community services provided by hospitals to impact public health.

## 1. Introduction

As of March 2010, Internal Revenue Service (IRS) requires not-for-profit hospitals to detail their community benefit programs in order to justify tax-exempt status [1]. Specifically, all tax exempt hospitals are to complete a tri-annual community health needs assessment (CHNA) and then provide responsive community benefit services. Frameworks are being offered to assist hospitals in this work [2]–[4], however, strategies and tools for implementing, evaluating, and sustaining these efforts have not been fully developed. To maximize the impact of these efforts, prevention services must be assessed before they can be incorporated into a standard of care resulting in widespread adoption by hospitals.

Whereas hospitals are accustomed to assessing needs for clinical services they are less prepared to consider foundation for their outreach work. With an increased focus on community-based prevention in general, hospitals will be best served by adopting approaches for assessing the degree to which they are providing these services and the extent to which these services are based on existing evidence-based approaches. With limited resources, hospitals will be increasingly motivated to direct resources to maximize impact.

Hospital-based injury programming can provide insight into this work. As injuries are the leading cause of morbidity and mortality in children and countermeasures exist to reduce this burden, many well-established and evidence-supported injury prevention programs have been supported by hospitals. For example, the Injury Free Coalition for Kids is exclusively house and delivered through Trauma 1 healthcare systems [5] and many hospitals lead their National Safe Kids chapters, which promotes the use of car seats and other approaches. In 2010, child car seats and booster seats were estimated to have saved 285 and 12,546 lives, respectively [6]. Economic analyses indicate that child safety seat distribution programs costs $55 and saves $2,200 in total costs per seat [7]. Further impetus for healthcare based injury prevention is supported in the 2012 National Action Plan for Childhood Injury Prevention from the Centers for Disease Control and Prevention (CDC). The plan emphasizes broad priorities for health care settings such as increasing awareness, seeking out evidence-based solutions, providing effective and relevant health information to the public, and supporting coordinated service efforts [8]. The action plan further highlights centralized coordination of programs which are most often provided by children's hospitals and trauma centers [9]–[11].

While U.S. children's hospitals are both encouraged and mandated to provide community outreach for injury prevention, the extent of this programming has not been previously described. This manuscript provides a description of the community-based injury prevention services offered by U.S. children's hospitals using a comprehensive assessment that is easily translated to other services. This assessment is the first step in establishing a standardized approach for community-based preventative healthcare efforts. Informed by the dissemination and implementation literature [12]–[17], the paper seeks to illuminate organizational factors related to the adoption of community outreach programming targeting injury prevention across a nationwide sample.

## 2. Materials and Method

### 2.1. Methods

Data were collected in partnership with the Children's Hospital Association. The Association is a national membership organization to which 95% of all U.S. children's hospitals belong. The Association works to enhance child health through innovation in the quality, cost, and delivery of healthcare. Members are primarily not-for-profit hospitals that provide short-term clinical care for children or specialty hospitals, such as those providing burn or orthopedic care to children. The Children's Hospital Association maintains a database of member hospitals, has identified the injury prevention specialist at each hospital, and conducts annual and topical surveys of its members. The members of the Children's Hospital Association are highly engaged in the work of the Association and receive regular reporting of results. All 232 member hospitals were invited to participate.

Recruitment consisted of a three-tier process. First, the Children's Hospital Association identified one contact person at each hospital as the injury prevention specialist (IPS), the person most involved in injury prevention services at their hospital and likely to be the most knowledgeable about the programming information included in the survey. Each was sent an email invitation for an online survey after confirming their identity as being appropriate. The survey was administered from May to July 2011. Participants completing the survey were entered into a lottery to win one of five trip incentives to attend Children's Hospital Association's 2012 Creating Connections Conference (a $1,500 value). Four follow-up emails were made to non-respondents. The survey was conducted using Survey Center, an on-line survey management tool supported by the Children's Hospital Association.

As it was our goal to obtain information from the location most central to injury prevention efforts, participants were asked to respond to the survey based on their personal knowledge of the hospital's injury prevention activities. Although many hospital members might have been involved in injury prevention work, participants were not asked to consult with others, due to the substantial respondent burden that would entail. All data were de-identified for purposes of analysis. This project was approved by the Saint Louis University Institutional Review Board.

### 2.2. Measures

Survey content was informed by previous Children's Hospital Association assessments in order to provide consistent benchmarking data. To the extent possible, items were modified from established, previously validated surveys developed from implementation science, diffusion of innovations, and evidence-based public health [18]–[25]. With a few exceptions, all items had closed-ended response options, and allowed for skip patterns as appropriate. Items were carefully reviewed for ambiguity and respondent burden and non-critical items were omitted from the final questionnaire. The questionnaire was evaluated by members of the target audience using a modified cognitive response testing technique to establish face and content validity.

The instrument contained six sections and took approximately 30 minutes to complete: About You (19 items, e.g., years in current position, education level), Injury Prevention at Your Hospital (11 items, e.g., what department houses the injury prevention program, other departments' support of injury prevention programming), Programs and Partnerships (40 items; specific partners involved with injury programming and what topics and age groups are addressed by hospital programming), Making Program Decisions (42 items; how data are used to make decisions, importance of various factors when deciding about program delivery), Staffing, Budget and Resources (18 items; e.g., FTEs and budget allocation) and Hospital Culture (18 items; injury prevention as part of the hospital's mission and the expectation of evidence based practice). The full assessment is available from the corresponding author.

### 2.3. Data analysis

Descriptive analyses were conducted using SPSS version 18 [26]. Frequencies are presented for categorical responses and means, medians and ranges are reported for continuous measures.

## 3. Results

The overall response rate was 58%, corresponding to a sample of 135 injury prevention specialists representing children's hospitals from 39 different states in the US.

### 3.1. Hospital demographics

Of the 135 respondents, 60.7% were described as children's hospitals within larger hospitals, 26.7% were free-standing children's hospitals, and 12.6% were specialty children's hospitals. This response distribution approximates the composition of all Children's Hospital Association member hospitals (63%, 20%, and 17%, respectively) and is similar to the distribution of non-respondents (65%, 13% and 22%, respectively). Regarding size, the average number of beds of responding hospitals was 163, similar to the mean number of beds of member hospitals (155).

Fifteen percent of respondents reported that the majority of their service population is rural, 44% reported a majority urban and 21% indicated their service majority was suburban. The remaining 20% reported that their service population was the same for rural, urban and suburban. Seventy three percent of hospitals are verified as a pediatric or adult level trauma center, which requires injury prevention activities for accreditation purposes.

### 3.2. Injury prevention programming administration and offerings

The majority of responding hospitals' injury prevention programming units are well established and fairly autonomous with 60.3% considered their own unit (i.e., not subsumed under larger departments or decision-making structures) and 71.8% responsible for managing their own budgets. Eighty five percent indicated that they had been in operation for three or more years. Regarding program leadership, 35.9% had a director of programming, 38.2% had a medical or clinical director, and 22% had both.

Of the responding hospitals, 95% reported offering pediatric injury prevention programming. For a majority (64.1%) of the hospitals, the injury prevention programming was administratively located in the children's hospital itself. For 29% of hospitals, the programming was housed in the larger healthcare system with or without ties to the children's hospital. Although this describes the central administrative location of injury programming, over half (55.7%) of respondents reported that multiple areas offer such programming. Fifty-three percent of respondents indicated that the programming home base was clinical (e.g., emergency medicine, trauma, or pediatrics), 39% indicated non-clinical (e.g., marketing, advocacy, or community outreach), and 9% considered their home base shared between clinical and non-clinical departments.

**Figure 1.**
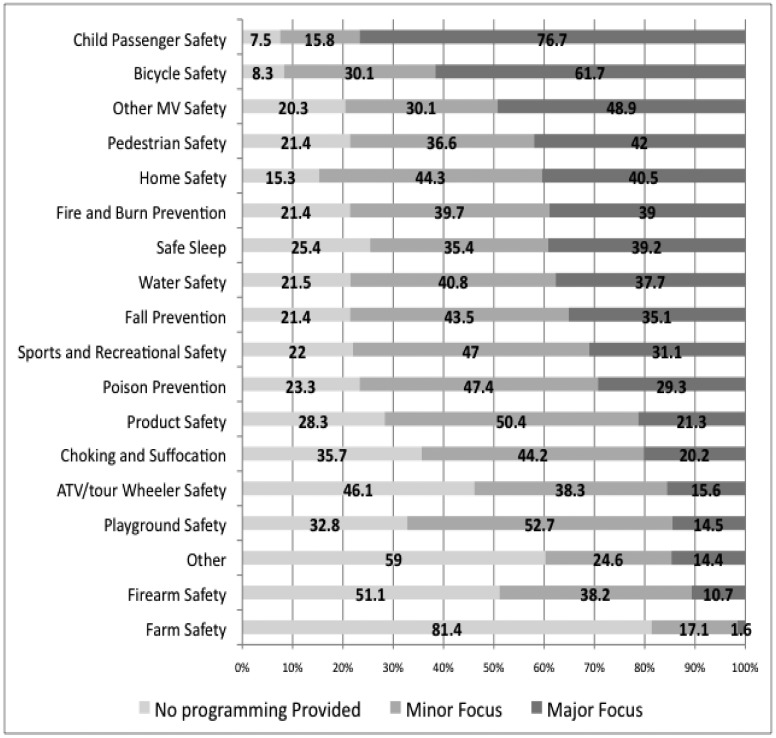
Percentage of responding hospitals that reported programming area was a major or minor focus or that no such programming was provided for selected IP topics (*n* range 61-133).

When asked to identify the areas in which programming was offered, child passenger safety was the primary major focus of responding hospitals as a group, with 76.7% of hospitals indicating that this was a major focus and 15.8% indicating a minor focus (Figure 1). Other priority areas included bicycle safety and other motor vehicle safety (e.g., occupant safety, distracted driving, unattended and back-over, teen driving). Similar information was elicited about specific age groups served by injury prevention programming. A majority of hospitals had a major focus on infants (69.7%), preschoolers (73.5%), and elementary school age groups (79.5%). When asked to indicate the percentage of resources provided to patients and their families as compared to the external community, hospitals indicated a median of 34.5% directed to patients and their families and 63.3% directed to the external community. Only 18.8% indicated that their hospital operates a safety resource center (aka safety store), a dedicated space in the hospital to sell safety devices, usually at a discounted cost, and deliver corresponding health education.

### 3.3. Partnerships to address injury prevention

Approximately 70% of injury prevention specialists indicated that they were members of one or more professional organizations. Just over 50% are members of a professional organization for a particular clinical specialty (such as Pediatrics or Emergency Medicine), while membership in nationally based organizations such as American Public Health Association, Society for the Advancement of Violence and Injury Prevention Research, and Safe States Alliance ranged from 8% to 15%. Partnerships with national childhood injury prevention organizations were also prevalent among respondents (Table 1). Fifty-four percent of respondents reported that they had worked with their Safe Kids state chapter in the last 12 months, and 55% acted as the lead agency in their Safe Kids local chapter. Nineteen percent acted as the lead agency for Injury Free Coalition for Kids while 12% had worked with them within the last 12 months. Only 2 respondents indicated having no partnerships at all.

**Table 1.** Percentage of responding children's hospitals that have various levels of partnerships with injury prevention groups.

Organization	Leads local efforts in partnership with this national group	Worked with this group in the last 12 months	Not worked with the group in the last 12 months
Safe kids local chapter (*N* = 126)	54.8	27.8	17.5
Injury free coalition for kids (*N* = 122)	18.9	12.3	68.9
Safe Kids state chapter (*N* = 125)	17.6	54.4	28
Child death review board (*N* = 126)	8.7	51.6	39.7
Poison control center (*N* = 126)	7.9	56.3	35.7
Schools (*N* = 129)	*	95.3	4.7
Public Safety (*N* = 129)	*	93.0	7.0
Health/Social Services (*N* = 128)	*	85.2	14.8
State/Local Trauma (*N* = 129)	*	82.9	17.1
Other Hospitals (*N* = 127)	*	81.1	18.9
Other Academic Organizations (*N* = 126)	*	77.0	23.1
Non-government Agencies (*N* = 129)	*	65.9	34.1
Parks (*N* = 128)	*	64.1	35.9
Other (*N* = 57)	*	61.4	38.6

* Hospitals may partner with this organization locally, but may not lead.

### 3.4. Injury prevention resources

Sixty-one percent of respondents indicated they had an injury prevention budget, with a mean of $287,080 and median of $200,000. Budgets, including staff costs, ranged from $0 to $1.5 million. For the 56% of specialists reporting that other hospital groups offer injury prevention services, 34% report that other units have budgets for injury prevention work. Hospitals with IP budgets were more than twice as likely to receive revenues compared to those hospitals without IP budgets (89% vs. 41%; χ^2^ = 34.9; *P* < 0.0001). Altogether, seventy-one percent responded they received revenue (internal or external), with a mean of $221,268 and median of $125,000. The range of revenue received was similar to the budget range, from $0 to $1.4 Million. Overall, 38.3% of revenue received came from private funding, 22.5% from federal and state grants, 25.7% from hospital support, and 4.8% from payment for hospital services or product sales.

On average, hospitals had 3.64 full time equivalents (FTEs) dedicated to injury prevention, with a median of 1.6. These FTEs tended to be program staff rather than directors, researchers, clinical experts or administrative support. Forty-six percent of hospitals reported that the staff time devoted to injury prevention had increased over the past three years, while 43.4% reported a decrease in staff time. A large majority, 86%, utilized volunteers.

**Figure 2.**
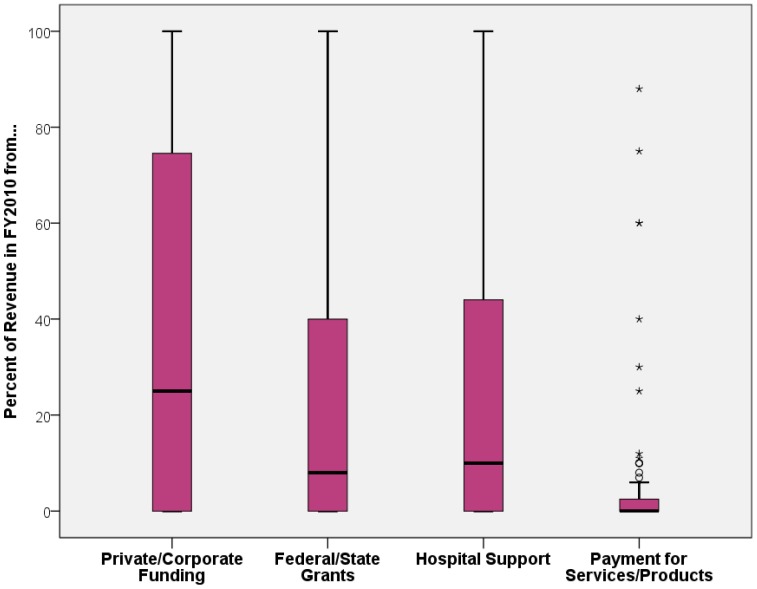
Box plot of the distribution of revenue sources.

The majority of funding for IP programming comes from private or corporate sources and federal and state grants (Figure 2). Hospital injury prevention programs are receiving proportionally less support from the hospital itself and payment for service or product sales. When asked if other units in the hospital also had an injury prevention budget, 32.1% said yes, 37.4% said no, and 30.5% were unsure.

Regarding the utilization of injury prevention activities for internal needs, 47.7% of hospitals request financial data from injury prevention programming to report as community benefit through IRS form 990 schedule H, 23.1% do not, and 29.2% were unsure of whether or not their hospital requests this information.

### 3.5. Selected comparisons

Comparisons by hospital type revealed statistically significant differences for FTEs, percent reporting program information for community benefit purposes, the number of major focus areas, the percent that have a budget and the mean budget (Table 2). No differences were observed across hospital type for the percent of hospitals that support IP programming. Further, the distribution of the number of injury prevention areas with a major focus did not significantly differ by hospitals with and without a trauma center (median number of major focus areas was 5 and 6, respectively).

**Table 2.** Comparison of selected measures by hospital type.

Hospital type	Have IP programming percent (overall *n*)	IP FTEsmedian (*n*)	Have IP budget percent (*n*)	IP Budgetmedian	Number of major IP focus areas median ( range)	IP for community benefit percent (*n*)
Children's hospitals within larger hospitals	92.7% (82)	1.6 (79)	56.4% (78)	$134,500.00	6 (1–14)	37.5% (80)
Free-standingchildren's hospitals	100% (35)	5.0 (33)	88.2% (34)	$340,000.00	5 (2–15)	82.9% (35)
Specialty children's hospitals	94.1% (17)	0.5 (16)	25% (16)	$21,000.00	2 (1–11)	20.0% (15)
Test for difference	*P* = 0.244	*P* < 0.0001	*P* < 0.0001	*P* < 0.0001	*P* < 0.0001	*P* < 0.0001

Differences in percentages across hospital types were conducted using Fisher's exact test. Comparisons of distributions across hospital type for FTEs and Budget values were conducted using the Kruskal Wallis Test.

## 4. Discussion

Results of this national survey provide a baseline assessment of injury prevention outreach undertaken by U.S children's hospitals and illustrate an approach for assessing characteristics that have been related to the provision of community outreach. The assessment approach can be used to examine other areas of public health programming. Our findings indicate that a majority of respondents report key characteristics that support evidenced based injury prevention programming: the presence of FTEs, areas of major programming in priority areas (such as motor vehicle crashes), and a strong administrative infrastructure. Approximately half of hospitals support staff salary and/or resources to assist with community based childhood injury prevention interventions and projects.

In order to advance evidence-based programming, hospitals must have the ability to partner with other organizations and individuals. Our research indicates that the majority of responding hospitals are collaborating often and partnering routinely with other organizations. These partnerships are particularly robust in the public domain, as almost all responding hospitals indicated partnerships with schools and public safety groups. Though fewer hospitals reported partnerships with professional/private organizations, a majority still maintains these ties. Therefore, the results highlight the need to focus nationally on developing and supporting strategic partnerships between local communities and both national organizations and other professional groups to further aid in program implementation. Such partnerships might provide venues for translating public health research into practice, technical assistance for program evaluation, and organizational support for program sustainability. For example, given that a large percentage of hospitals partner with schools, an initiative with the state departments of public instruction to support hospital and school collaborations for injury prevention servicing may be particularly successful. Importantly, these efforts would require on-going evaluation using longitudinal assessment approaches.

Using a comprehensive survey instrument, the results indicate that there is substantial variability between hospitals with respect to administration; while it is unclear how this variability affects injury prevention health services offerings, it may in part explain some of the variation in hospital priorities and how the hospitals are positioned to support non-traditional programming. Since there is no common “home base” for injury prevention programming and many hospitals have administrative leadership divided across several areas, injury prevention activities may become diffuse within an organization, or different priorities may compete for resources. Because injury prevention is broadly defined and covers topics ranging from infant safe sleep to teen driving, this is somewhat expected. Professionals from many different specialties will be involved and may potentially drive programming in different areas even across similar organizational systems. However because outreach efforts in general will likely be multidisciplinary, hospital outreach efforts in response to community health needs assessments may be similarly fragmented.

This variability in administrative structure may also be due to the large amount of federal and private funding secured by hospitals that tends to support specific priority topics or age groups, leading to further partitioning of injury prevention efforts. Therefore, as well-articulated in the National Action Plan for Child Injury Prevention [8], it may be beneficial for the national injury prevention agencies to move forward with a unified priority agenda for supporting injury prevention specialists in clearly defining local priorities and creating a more centralized approach to injury prevention across departments. Currently, absent changes in funding mechanisms, children's hospitals may prioritize organizational structures that identify a central umbrella for injury prevention outreach in order to best utilize internal resource to improve public health indicators.

Financial and staffing resources also vary widely across responding hospitals. A large portion of hospitals do not receive revenue (29%) or have a budget (39%) for injury prevention outreach. Of those that do, there is enormous variability in funding. Similar variability is seen in the number of FTEs dedicated to injury prevention; while most hospitals have very few FTEs, others have extensive teams. Developers of injury prevention interventions must consider the hospital resources required for implementation and sustainability and also the priority placed on local injury prevention efforts. While some hospitals may be well positioned to carry out complex programming, others may not have this capacity or goal. If this organizational capacity can be routinely measured in an assessment tool, advocates for such programming can better substantiate the need for internal support. Findings from this research can inform future service recommendations similar to the work in child abuse prevention [27] and efforts describing the capacity of organizations to perform specific evidenced-based functions in public health [28]. Collectively, such research is designed to reduce the impact of specific health threats and to garner support for those topics. Sustainable, evidence-based programming will be an important factor in developing a stronger capacity for hospitals to reduce the burden of injuries in local communities. Importantly, the injury prevention field must work to ensure that the burden of injuries is appropriately reflected in the Community Health Needs Assessments (CHNAs) and the Community Benefit Plans of not-for-profit hospitals. As national efforts drive healthcare entities toward prevention service to improve public health, it may be useful to develop classifications of the level of preventive care offered by hospitals, similar to the level of clinical service provided. Our data demonstrate sufficient variability for this purpose and can contribute to such discussions.

## 5. Conclusion

While the responses were provided by the hospital representative identified as being the most knowledgeable about injury prevention efforts, the responses represent only one person's view of injury prevention programming—an important limitation since programming occurs throughout the organization. Future work might examine the variation in reporting across hospital representatives. As with all surveys, there is a possibility of item response bias as participants may have wanted to portray their hospital and its programs in a more positive light. Non-response bias is also possible given that 43% of Children's Hospital Association members did not respond. However, this bias is likely minimal since the distribution of hospital type, size and geography are similar for responding and non-responding hospitals. The response rate is also similar to other surveys of hospitals [29]. This assessment represents the integration of injury prevention, dissemination and implementation and community health needs assessment in order to understand an area of hospital outreach. Instrument quality and measurement development work should be continued as this area of research is developed. Further research on individual components impact on programming may further guide the organizational structure of injury prevention programs to maximize the impact on community injury rates.
